# Genome-Wide Identification and Functional Analysis of Lysine Histidine Transporter (LHT) Gene Families in Maize

**DOI:** 10.1155/2022/2673748

**Published:** 2022-04-26

**Authors:** Md. Golam Rabby, Md. Munnaf Hossen, Md. Mostafa Kamal, Md. Numan Islam

**Affiliations:** ^1^Department of Nutrition and Food Technology, Jashore University of Science and Technology, Jashore 7408, Bangladesh; ^2^School of Medicine, Shenzhen University, Shenzhen, Guangdong 518060, China

## Abstract

Amino acid transporters (AATs) are essential membrane proteins that transfer amino acids across cells. They are necessary for plant growth and development. The lysine histidine transporter (*LHT)* gene family in maize (*Zea mays*) has not yet been characterized. According to sequence composition and phylogenetic placement, this study found 15 *LHT* genes in the maize genome. The *ZmLHT* genes are scattered across the plasma membrane. The study also analyzed the evolutionary relationships, gene structures, conserved motifs, 3D protein structure, a transmembrane domain, and gene expression of the 15 *LHT* genes in maize. Comprehensive analyses of *ZmLHT* gene expression profiles revealed distinct expression patterns in maize *LHT* genes in various tissues. This study's extensive data will serve as a foundation for future *ZmLHT* gene family research. This study might make easier to understand how *LHT* genes work in maize and other crops.

## 1. Introduction

Maize (*Zea mays*) is a significant and widely produced crop throughout the world. Maize is vital for food and nutritional security as well as economic viability, particularly in resource-scarce smallholder communities [1]. Additionally, it is one of the world's most significant cereal crops and serves as a model system for genetic research [2]. Maize relies heavily on both inorganic (nitrate and ammonium) and organic (amino acids, peptides, and proteins) nitrogen sources in the soil/media [3, 4]. Nitrogen (N) is a critical nutrient for plant growth since it is required in a range of compounds in various forms and is primarily found in plants as amino acids. Amino acids are necessary for the formation of enzymes and proteins and the metabolism and structure of plants [5]. Inorganic nitrogen (nitrate and ammonium) can be converted in roots and leaves to amino acids [6]. Proteolytic enzymes require amino acids (AAs) for their production. Amino acids also create polyamines, nucleotides, creatinine, and glucosamine [7]. Furthermore, they are the precursors of nucleic acids, phytohormones, and chlorophyll. They are also called the low-nitrogen transfer modes [8]. They are also precursors of secondary metabolites, which are essential for plant growth and development. Abiotic and biotic stresses are also discussed concerning amino acids [9]. Plants get nitrogen from amino acids and uptake from soil by the roots and transport to leaves as well as to other organs via the phloem. The xylem carries amino acids in roots. Membrane transporter proteins are necessary for root cells to uptake amino acids [10].

AATs are membrane proteins that transport amino acids across cell membranes in higher plants. Amino acid transport and absorption from soil are also regulated by these enzymes [11]. The AAT gene family has been identified in many plant species. Examples of the AAT gene family's breadth include *Arabidopsis* (63), rice (85), soybean (189), and potato (72) [12–15]. AATs are classified as AAAP (amino acid/auxin permease) or APC (amino acid-polyaminecholine) transporters. AAAP family includes amino acid permease (AAP), *γ*-aminobutyric acid (GABA) transporter (GAT), lysine/histidine transporter (LHT), like-auxin influx carriers (LAX), proline transporter (ProT), aromatic and neutral amino acid transporter (ANT), vesicular aminergic-associated transporter (VAT), and amino acid transporter-like proteins (ATL) (VAAT). APC family members are further divided into four subfamilies: amino acid/choline transporters (ACT), cationic amino acid transporter (CAT), tyrosine-specific transporter (TTP), and polyamine H+ cotransporters (PHS). In addition, the AAT superfamily now includes a new subfamily called Usually Multiple Amino Acids Move in and Out Transporters (UMAMIT) [16, 17]. The presence and quantity of amino acids in the rhizosphere determine the kind of transporter engaged in root uptake.

Candidates could be from the LHT family, described as a lysine and histidine selective transporter which includes high affinity neutral and acidic amino acid transporters [18]. Studies on LHT members revealed their ability to transport a broad spectrum of amino acids. *LHT* genes have been found in roots, leaves, and flowers. They can mediate amino acid uptake from the soil and transport and partition inside plants [19]. According to functional tests of 53 putative *Arabidopsis* AATs, LHT1 has a higher affinity for amino acids than AAP subfamily transporters. In *Arabidopsis*, a single LHT1 mutation stops plant growth and affects amino acid intake and distribution. To better understand the N cycle in plants, we may overexpress LHT1 in nitrogen-scarce plants [20, 21]. AtLHT6 is required for the uptake of acidic amino acids, glutamine, alanine, and maybe phenylalanine by root cells [18]. Ten *Arabidopsis LHT* genes are involved in AA import into tapetum cells, trichomes, and have a role in organic N transfer for pollen production [22].

There are six *Arabidopsis* LHT transporters found in male and female floral tissue: AtLHT1, 2, 4, 5, and 6. They are also needed for proper sexual plant reproduction. Also, AtLHT4 and AtLHT7 may be involved in anther and pollen development [23]. A qRT-PCR investigation identified OsLHT1 expression in rice root, stem, flag leaf, sheath, and immature panicle. OsLHT1 is restricted to the plasma membrane in rice protoplasts, consistent with its amino acid transporter activity. The loss of OsLHT1 function significantly reduced rice growth and fertility [19].

LHTs' strong affinity and substrate selectivity for neutral and acidic amino acids were likely due to their gene function in plant progenitors. Studies on *Arabidopsis* LHT localization and expression suggest that LHTs are important in seed plant sexual reproduction [23]. Phylogenetic research guides the evolutionary genesis of amino acid transporters in maize [5, 24]. Phylogeny and conserved domains identified genes with similar biological roles and close evolutionary links [25–28]. Its functional structure is determined by the number of introns in a gene, and the transmembrane helices decide on its tertiary structure in a protein [25, 29].

The goal of this study was to do a genome-wide identification and phylogenetic analysis of *ZmLHT* genes and investigate the evolution of this gene family. In addition, the characteristics of exon-intron structures, patterns of conserved motifs, 3D protein structures, transmembrane domains, and expression patterns were also investigated. The information gathered will aid research into the biological functions of the *LHT* gene family in maize.

## 2. Materials and Methods

### 2.1. Identification of the ZmLHT Gene Family Members in Maize

The LHT proteins were found in maize using an HMM (hidden Markov model) profile of AtLHT protein conserved domains based on the PFAM, CDD, and SMART databases. Briefly, a local database based on protein sequences was generated using the TBtools software after downloading the maize genome (B73 RefGen v4) sequence from the maize database (http://www.maizesequence.org/index.html). The known fifteen *Arabidopsis* LHT protein sequences were downloaded from TAIR (http://www.arabidopsis.org) and utilized as queries in a local BLASTP search against the maize protein database with an e-value of 1e-5 and a criterion of up to 50% identity. Furthermore, all *Arabidopsis* LHT protein sequences were aligned for multiple sequence alignment using ClustalX 2.1 (https://clustalx.software.informer.com/2.1/), and the results were used to search the maize database using the online HMMER search engine. Manual editing was used to compare and parse the results of the HMMER search tools and BLASTP. The PFAM database (http://pfam.xfam.org/), the NCBI conserved domain database (CDD) (https://www.ncbi.nlm.nih.gov/cdd/), and the SMART database (http://smart.embl-heidelberg.de/) were used to eliminate redundant sequences by testing for the presence of ZmLHT (PF01490) conserved. To confirm the conserved domains in the remaining sequences, Inter-Pro-Scan (http://www.ebi.ac.uk/interpro/scan.html) was utilized. These designations were given to maize genes that were close to *Arabidopsis* genes. The physical and chemical properties of the ZmLHT proteins were examined using the ProtParam service (http://web.expasy.org/protparam/). To estimate the subcellular localization of ZmLHT proteins, the ProtComp server (http://linux1.softberry.com/) was utilized [11, 30, 31].

### 2.2. Phylogenetic Tree Construction

The ZmLHT protein sequences generated from the maize genome were aligned using ClustalX (version 2.1). The phylogenetic tree of ZmLHT proteins was then generated using the MEGAX software by comparing AtLHT proteins using the maximum likelihood (ML) technique and 1000 bootstrap replicates [11, 30].

### 2.3. Gene Structures and Conserved Motifs Analysis

The web-based GSDS server (http://gsds.cbi.pku.edu.cn/) was used to investigate the gene structure of ZmLHT proteins, and the conserved domains of the ZmLHT proteins were characterized using the Pfam (http://pfam.xfam.org/) and TBtools software. The MEME suite (version5.0.5) (http://meme-suite.org/tools/meme) was used to determine the number of conserved motifs based on optimum E-values [30].

### 2.4. Prediction of Three-Dimensional Modeling

The Phyre2 website (http://www.sbg.bio.ic.ac.uk/phyre2) was used to create the three-dimensional structure of representative ZmLHT proteins in order to investigate structural modifications and their effects on the functions of maize ZmLHT family members [11].

### 2.5. Transmembrane Domain Prediction

The crystal structures of transmembrane topology were constructed using the online tool HMMTOP (http://www.sacs.ucsf.edu/cgi-bin/hmmtop.py), and the transmembrane helices of the *ZmLHT* proteins were calculated manually [32].

### 2.6. Gene Expression Analysis

For the expression-based classification of *ZmLHTs*, gene expression data from a variety of organs at various stages of development, including the leaf, internodes, root, reproductive tissues (tassel, silk, and cob), seed, embryo, and endosperm were analyzed. Their gene expression data retrieved from MaizeMine (http://maizemine. rnet. missouri. edu: 8080/maizemine/begin.do), a publicly accessible transcriptome resource [33]. The heat map was created using log2 transformed FPKM (fragments per kilobase per million) values to show expressions. The FPKM values were determined using Edge R, an R program. The TBtools software generated the heat maps for the FPKM values dataset [34].

## 3. Results

### 3.1. Identification and Characterization of the Members of LHT Gene Families in Maize

A total of 15 LHT proteins were identified in the maize genome by sequence validation and designated ZmLHT1-ZmLHT15 (Table 1). The biochemical and physiological characteristics of all translated proteins are summarized in Table 1. The amino acids length in maize LHT proteins ranged from 160 (ZmLHT15) to 904 (ZmLHT1). The molecular weights of the ZmLHT proteins ranged from 18.27 (ZmLHT15) to 100.65 (ZmLHT1) kD. The pI values of all ZmLHT proteins exceeded 7, indicating that they were all basic proteins. GRAVY scores of all proteins were positive, indicating that they were hydrophobic. All ZmLHT proteins are expected to be localized to the plasma membrane on a subcellular level.

### 3.2. Phylogenetic Analysis of the Members of LHT Gene Families in Maize

**Figure 1:**
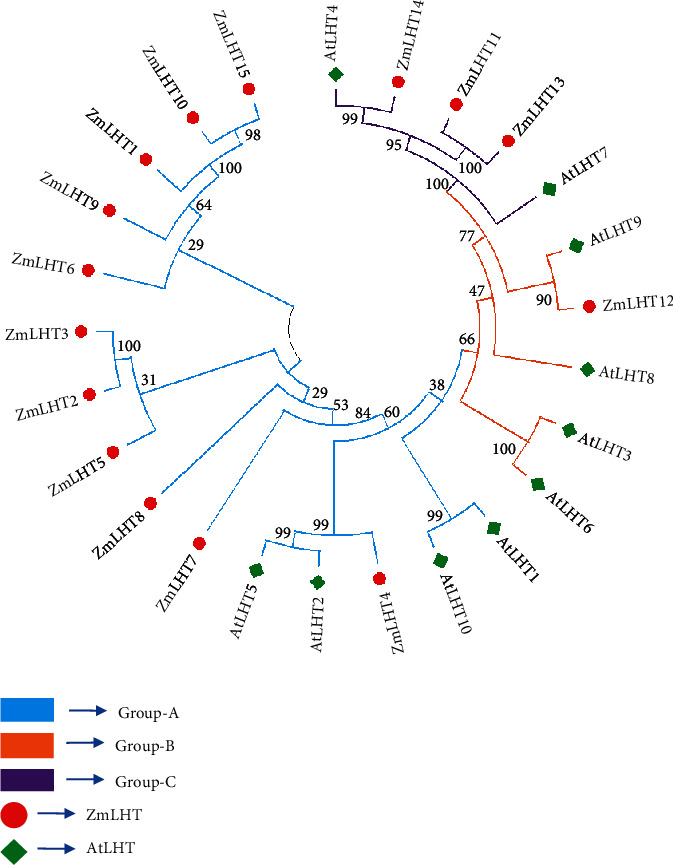
Analysis of phylogenetic tree of *LHT* gene family in maize.

Based on the results of phylogenetic analysis, 15 LHT proteins were classified into three subcategories (Figure 1). The three subcategories are A, B, and C. Group A included 11 ZmLHTs (1–10 and 15). Only ZmLHT12 was classified as a member of group B, whereas the remaining three ZmLHTs (11,13, and 14) were classified as members of group C. Maize LHT proteins belonging to subclasses A, B, and C may share functional similarities with AtLHT1,2,5,10; AtLHT3,6,8,9; and AtLHT4,7 proteins; respectively. The HMM model's encoded genes did not agree with the phylogenetic analyses. This could be because the algorithms utilized in the two models are different. Both, however, account for the functional characteristics of the LHT proteins, which are connected with distinct clades of Arabidopsis genes.

### 3.3. Gene Structures and Conserved Motif Analysis of LHT Gene Families in Maize

**Figure 2:**
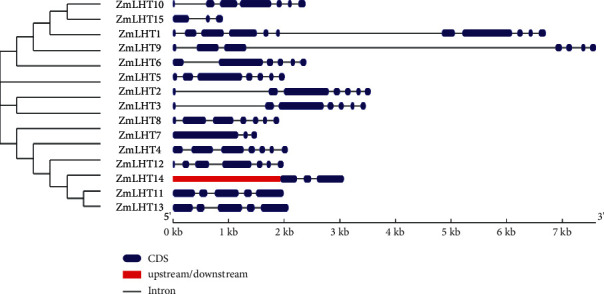
Analysis of exon-intron structure of *LHT* gene family in maize.

The emergence of multigene families has had a significant impact on the diversity of gene architecture. Exon-intron structures vary among ZmLHTs, as illustrated in Figure 2. Under different cluster groups, the number of introns in the *ZmLHT* gene family varies from two (ZmLHT2,14, and 15) to ten (ZmLHT1) (Figure 2). The number of introns in ZmLHT group A ranges from two (ZmLHT2,14) to ten (ZmLHT1). As a member of group B, the ZmLHT12 has six introns. The number of introns in ZmLHT group C varies from two (ZmLHT2) to four (ZmLHT11,13). The intron-exon organization and phylogenetic tree demonstrated that members of the same group had similar gene structures.

**Figure 3:**
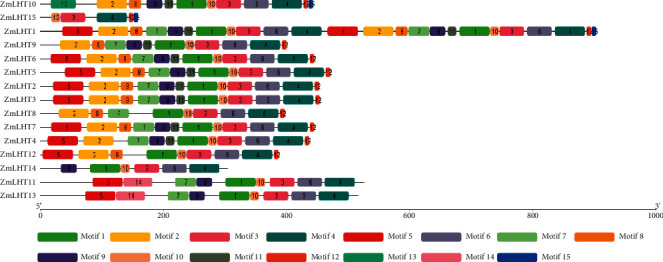
Analysis of conserved motif of *LHT* gene family in maize.

Motif analysis resulted in the identification of total 15 motifs, labeled as 1 to 15 (Figure 3). ZmLHT proteins have anywhere from five (ZmLHT15) to 13 (ZmLHT1) motifs. Except for ZmLHT14 and 15, all members of the ZmLHT protein family had motif 1, 3, 4, 6, and 10 in common. All proteins in group A had the motifs 1, 2, 3, 4, 6, 10, and 12; nine motifs (motifs 1–6, 8,10, and 12) were found in all proteins in group B; and six motifs (motifs 1, 3, 4, 6, 9, and 10) were identified in all proteins in group C. Furthermore, the ZmLHT 2, 3, 5, 6, and 7 proteins all had the same number (12) of motifs (1–12) (Figure 3).

### 3.4. Three-Dimensional Modeling of the LHT Gene Family in Maize

**Figure 4:**
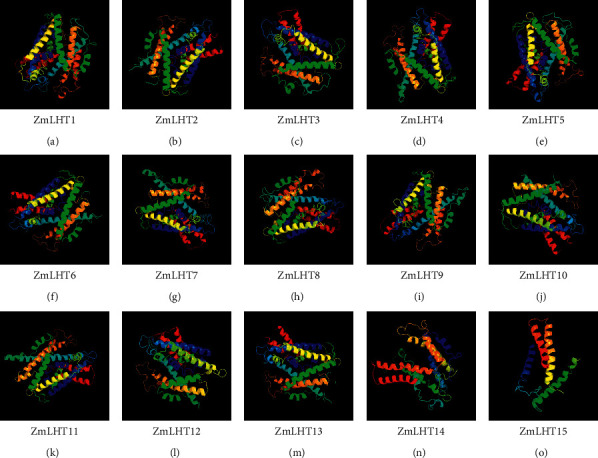
Analysis of three-dimensional protein structure of *LHT* gene family in maize.

After predicting the gene structures of ZmLHTs, we analyzed secondary protein structures to predict the rearrangements of structures and the nature of polypeptide bonds present in ZmLHTs proteins. As per three-dimensional protein structure analysis, all ZmLHT proteins had numerous alpha helices, transmembrane helix, and coil topologies (Figure 4). In the ZmLHTs, the percentage of alpha helix ranged from 63% (ZmLHT11) to 81% (ZmLHT15). The fraction of transmembrane helices in ZmLHTs ranged from 50% (ZmLHT11) to 68% (ZmLHT12). All proteins had a 100% confidence level (Table S1). On the other hand, in *Arabidopsis* LHT (AtLHT) protein, the percentages of alpha helix ranged from 64% to 72%. Compared to AtLHT, the percentage of alpha helix in ZmLHTs is higher up to 81% (ZmLHT15). However, the overall percentages of alpha helix are identical between AtLHT and ZmLHT proteins. Similarly, fraction of transmembrane helices in ZmLHTs and AtLHTs are identical to each other (Table S2), which summarized that the protein structure of ZmLHT and AtLHT are almost similar on the basis of secondary structure of proteins.

### 3.5. Transmembrane Domains in LHT Gene Family in Maize

**Figure 5:**
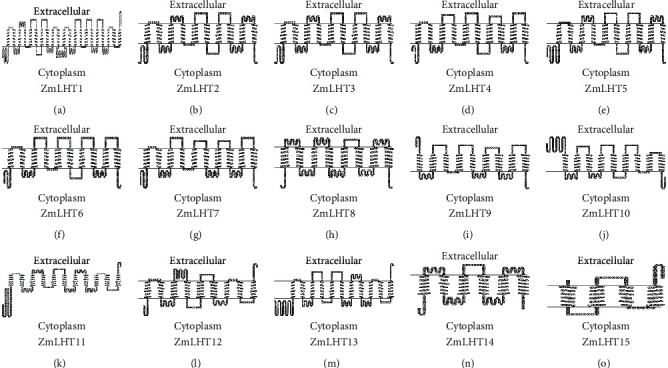
Analysis of transmembrane topology of *LHT* gene family in maize.

The ZmLHT proteins have multiple transmembrane (TM) helices in their transmembrane domains. Except for ZmLHT14 and 15, which had six and four transmembrane helices but extended intracellular sequences at the N-terminus and C-terminus, all LHT proteins had 8–12 transmembrane helices (Figure 5). Transmembrane segments were found to span the full length of all LHT proteins. The transmembrane domains of the ZmLHT proteins range in length from 160 (ZmLHT15) to 904 (ZmLHT1) (Table S3).

### 3.6. Expression Analysis of Maize LHT Genes in Different Tissues and Developmental Stages

**Figure 6:**
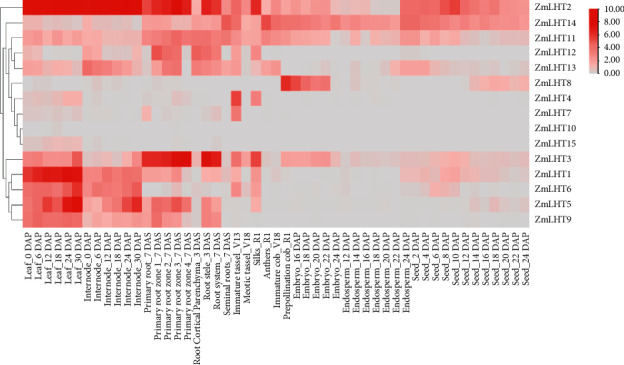
Clustering of maize LHTs based on gene expression. Heat map showing hierarchical clustering of genes based on gene expression in different tissues and at distinct developmental stages mentioned at the top. Clustering was based on log2 transformed FPKM values.

The expression patterns of *ZmLHTs* were investigated using RNA-seq data retrieved from an online database from specific tissues at distinct developmental stages. On the heat map, the normalized log2 (FPKM) value was displayed (Figure 6). Based on their expression patterns, the *ZmLHTs* were divided into three groups. Except for embryo and endosperm tissues, the single (*ZmLHT2*) gene in the first cluster exhibited a relatively high expression level and was stably expressed in almost all tissues at different developmental stages. The eight (*ZmLHT*4,7,8, and 11–15) genes in the second cluster were expressed in low amounts in most tissues where expression level of *ZmLHT10* is near to zero. However, a few, such as *ZmLHT*11 and 14, were slightly expressed in all tissues except seed and leaves, respectively, while *ZmLHT*13 were slightly expressed in primary root zones. The other five (*ZmLHT*1,3,5,6, and 9) genes in the third cluster showed a wide range of spatiotemporal expression patterns, with *ZmLHT1* and 5 being highly expressed in the leaves, and *ZmLHT3* being highly expressed in the roots. Furthermore, *ZmLHT1* was found to be slightly expressed in two or more tissues, such as leaves, internodes, and roots; *ZmLHT2* was found to be moderately expressed in leaves, internodes, roots, and reproductive tissues. On the other hand, *ZmLHT3* was found to be highly expressed in roots compared to leaves.

## 4. Discussion

Amino acid transporters (AATs) transport and distribute several types of amino acids in plants, and they are key targets for crop development [35]. AATs have been found in *A. thaliana, O. sativa,* and *S. tuberosum*, among other plant species. However, LHTs in Z. mays have yet to be discovered. In this study, 15 LHTs were identified in *Z. mays* that contained AAAP super families based on their resemblance to previously reported AATs in *Arabidopsis thaliana*, *O. sativa,* and *S. tuberosum* plants [36, 37]. Surprisingly, the number of members in the AAP subfamily is the same in *Arabidopsis, S. tuberosum,* and *S. lycopersicum* (8), but it is more than double in *O. sativa* (19 members). The increase of AAP subfamily members in *O. sativa* could be the result of tandem and segmental duplication events [36]. The total number of members in a same subfamily varies by species. For example, the LHT subfamily has six members in *O. sativa,* ten in *Arabidopsis,* 11 *in S. tuberosum,* 13 *in S. lycopersicum* [38], and 15 in *Z. mays* (Table 1). Different subfamilies showed an abundant variety in subcellular localization, such as AAP, LHT, AUX, GAT, and ProT were found on the plasma membrane, whereas TTP, ACT, ATLb, and ANT were located on the vacuole membrane. However, few members of the same subfamily displayed diverse subcellular localization, such as the ATLa subfamily genes positioned on both the plasma membrane and vacuole; the CAT and PHS subfamilies placed on the plasma, chloroplast, and vacuole [34]. In this study, all 15 *LHT* genes were located on the plasma membrane (Table 1).

Previous research recommended that motifs 1–4 function as a transmembrane region containing transmembrane transporter activity. The existence of the same type of conserved motif recommends that members of the same family may perform similar activities [39]. In this study, we found a total of 13 unique motifs and all ZmLHTs have at least 1–4 motifs except ZmLHTs11, 13, 14, and 15. Motif analysis revealed that the identified *ZmLHTs* exhibit transmembrane transporter activity. The multiple alpha helices structures during three-dimensional protein modeling guide the functional efficiency and evolutionary origin of a protein. The stable transmembrane helices indicate the functional integrity of proteins [33]. The analyzed three-dimensional structure of ZmLHT proteins with multiple alpha and transmembrane helices indicate the functional integrity and transport efficiency of the LHT proteins.

Transmembrane domain guides to evaluate the internal membrane protein structure and the full topology of protein. It also helps to predict genome analysis such as structural variation and gene expression [36]. The ZmLHTs hydrophobic transmembrane domain was predicted, and the family members had 4–21 transmembrane helices. The transmembrane structure can be classified into three categories based on the position of the N/C terminal: the N/C terminals of ZmLHT2–8 and 14 were all predicted as cytoplasmic and containing 6–10 TMs; the N/C terminals of ZmLHT15 were predicted as extracellular and containing 4 TMs; and the N/C terminals of the other ZmLHT proteins were predicted both as extracellular and cytoplasmic containing 9–21 TMs, including ZmLHT1 and 9–13. Our analysis results highlighted that all ZmLHTs are located in the plasma membrane that represents all LHTs might responsible for the transportation of amino acid from cell wall to inside the cell [23]. The study of *ZmLHT* gene expression patterns may yield important information for determining their likely function [40, 41]. The current study found that a number of genes were expressed in specific tissues and developmental stages based on RNA-Seq data analysis of ZmLHT genes. As shown in Figure 6, the expression profiles of *ZmLHT* genes that were found to be highly expressed may be linked to certain organs such as leaves, roots, internodes, and reproductive cells. According to prior research, AtLHT1 is essential for amino acid intake and mesophyll production in roots, whereas AtLHT2 is primarily involved in amino acid movement and allocation in floral organs [42, 43]. Another study discovered that AtLHT1 and AtLHT6 are highly expressed in roots and play a role in amino acid intake from the soil [44]. CsLHT1, CsLHT2, and CsLHT6 are likely involved in the root uptake of amino acids from the soil in the tea plant [45]. SiLHT2, 5, and 10 play critical roles in the transport and distribution of amino acids in the leaf; SiLHT11 uptakes amino acids in the root; and SiLHT9 participates in the long-distance transportation of amino acids from the root to the leaf in foxtail millet [34]. The current study's spatiotemporal expression analysis revealed that some of the discovered genes are selectively expressed in leaves, internodes, roots, and reproductive organs. ZmLHT2 is abundant in leaves, internodes, and roots. Furthermore, ZmLHT1, 3, 5, 6, and 9 are substantially expressed in leaves but only modestly so in internodes. In summary, the expression patterns suggest that *ZmLHTs* may play an important role in amino acid intake in various tissues and developmental stages of maize.

The current study's elucidation of the *ZmLHT* genes would pave the way for more research in AATs to better understand their importance and roles in plant physiology as well as in yield.

## 5. Conclusion

To summarize, this was the first comprehensive and systematic study of the *LHT* gene family in maize. The maize genome contains a total of 15 *ZmLHT* genes. Following that, in-silico analysis and expression analysis were used to deduce the probable functions of these genes in maize growth, development, and functional advancement. The findings indicate that the *ZmLHT* genes are involved in multiple aspects of nitrogen metabolism and might have an effect on maize growth and development. These findings pave the way for additional research into the molecular mechanisms behind the uptake, assimilation, and transport of nitrogen and amino acids in maize.

## Figures and Tables

**Table 1:** The characterization and identification of the ZmLHT family in maize.

Transcript ID	Gene Name	Amino acid (aa)	Protein molecular weight (kDa)	pI	GRAVY	Subcellular localization
Zm00001d035166_P001	ZmLHT1	904	100645.17	9.07	0.399	Plasma membrane
Zm00001d035157_P001	ZmLHT2	454	50423.55	9.14	0.494	Plasma membrane
Zm00001d049640_P002	ZmLHT3	455	50585.62	9.06	0.469	Plasma membrane
Zm00001d002176_P001	ZmLHT4	438	48418.33	9.16	0.580	Plasma membrane
Zm00001d035162_P001	ZmLHT5	472	51983.21	8.96	0.500	Plasma membrane
Zm00001d024204_P001	ZmLHT6	446	49653.49	9.09	0.473	Plasma membrane
Zm00001d037789_P001	ZmLHT7	446	49099.94	9.29	0.576	Plasma membrane
Zm00001d035161_P001	ZmLHT8	398	43768.95	9.04	0.616	Plasma membrane
Zm00001d035163_P001	ZmLHT9	401	44770.18	9.33	0.580	Plasma membrane
Zm00001d031922_P001	ZmLHT10	444	49486.96	8.59	0.380	Plasma membrane
Zm00001d002673_P001	ZmLHT11	527	56753.92	9.56	0.467	Plasma membrane
Zm00001d003403_P001	ZmLHT12	388	42052.52	9.40	0.561	Plasma membrane
Zm00001d026131_P001	ZmLHT13	517	55729.50	9.33	0.492	Plasma membrane
Zm00001d041004_P001	ZmLHT14	304	33255.19	9.20	0.667	Plasma membrane
Zm00001d021186_P001	ZmLHT15	160	18269.97	9.35	0.757	Plasma membrane

## Data Availability

The data which support the results of this study are available in databases described in the manuscript and from the corresponding authors upon request.

## Supplementary Materials

Supplementary Materials Supplementary table 1: characterization of three-dimensional modeling of the ZmLHT gene family in maize. Supplementary table 2: characterization of three-dimensional modeling of the AtLHT gene family in *Arabidopsis.* Supplementary table 3: prediction of transmembrane domain region of ZmLHT gene family in maize.
