# Supplementary material for: Genome-Wide Identification and Functional Analysis of Lysine Histidine Transporter (LHT) Gene Families in Maize
Source: Genet Res (Camb). 2022 Apr 26;2022:2673748. doi: 10.1155/2022/2673748 (PMC9064515; doi:10.1155/2022/2673748)
Supplement: Supplementary Materials — Supplementary table 1: characterization of three-dimensional modeling of the ZmLHT gene family in maize. Supplementary table 2: characterization of three-dimensional modeling of the AtLHT gene family in Arabidopsis. Supplementary table 3: prediction of transmembrane domain region of ZmLHT gene family in maize. [file 2673748.f1.docx]

**Supplementary Table.1** Characterization of Three-Dimensional Modeling of the ZmLHT Gene Family in Maize

| **Gene ID** | **Alpha helix**  **(%)** | **Beta strand (%)** | **TM helix**  **(%)** | **Disordered**  **(%)** | **Confidence**  **(%)** | **Coverage**  **(%)** |
| --- | --- | --- | --- | --- | --- | --- |
| ZmLHT1 | 68 | 0 | 57 | 20 | 100.0 | 41 |
| ZmLHT2 | 70 |  | 58 | 19 | 100.0 | 84 |
| ZmLHT3 | 71 |  | 58 | 19 | 100.0 | 84 |
| ZmLHT4 | 72 |  | 60 | 17 | 100.0 | 86 |
| ZmLHT5 | 69 |  | 57 | 22 | 100.0 | 81 |
| ZmLHT6 | 71 |  | 59 | 17 | 100.0 | 85 |
| ZmLHT7 | 71 |  | 59 | 18 | 100.0 | 85 |
| ZmLHT8 | 76 |  | 61 | 12 | 100.0 | 93 |
| ZmLHT9 | 76 |  | 61 | 14 | 100.0 | 91 |
| ZmLHT10 | 69 |  | 59 | 20 | 100.0 | 76 |
| ZmLHT11 | 63 |  | 50 | 28 | 100.0 | 72 |
| ZmLHT12 | 79 |  | 68 | 10 | 100.0 | 87 |
| ZmLHT13 | 66 |  | 52 | 27 | 100.0 | 73 |
| ZmLHT14 | 75 |  | 61 | 15 | 100.0 | 85 |
| ZmLHT15 | 81 |  | 57 | 14 | 100.0 | 82 |

**Supplementary Table 2.** Characterization of Three-Dimensional Modeling of the AtLHT Gene Family in *Arabidopsis*

| **Gene ID** | **Alpha helix**  **(%)** | **Beta strand (%)** | **TM helix**  **(%)** | **Disordered**  **(%)** | **Confidence**  **(%)** | **Coverage**  **(%)** |
| --- | --- | --- | --- | --- | --- | --- |
| AtLHT1 | 71 | 0 | 59 | 17 | 100.0 | 82 |
| AtLHT2 | 72 |  | 60 | 17 | 100.0 | 85 |
| AtLHT3 | 71 |  | 59 | 18 | 98.1 | 89 |
| AtLHT4 | 64 |  | 52 | 26 | 97.3 | 78 |
| AtLHT5 | 72 |  | 60 | 16 | 100.0 | 85 |
| AtLHT6 | 71 |  | 58 | 17 | 99.9 | 84 |
| AtLHT7 | 68 |  | 55 | 22 | 97.1 | 83 |
| AtLHT8 | 71 |  | 59 | 19 | 98.5 | 88 |
| AtLHT9 | 72 |  | 60 | 16 | 100.0 | 86 |
| AtLHT10 | 71 |  | 58 | 18 | 100.0 | 83 |

**Supplementary Table.3** Prediction of Transmembrane Domain Region of ZmLHT Gene Family in Maize

|  | **LHT1** | **LHT2** | **LHT3** | **LHT4** | **LHT5** | **LHT6** | **LHT7** | **LHT8** | **LHT9** | **LHT10** | **LHT11** | **LHT12** | **LHT13** | **LHT14** | **LHT15** |
| --- | --- | --- | --- | --- | --- | --- | --- | --- | --- | --- | --- | --- | --- | --- | --- |
| **TM1** | 58-77 | 39-58 | 39-58 | 30-53 | 58-77 | 35-54 | 36-59 | 16-40 | 22-39 | 81-99 | 112-129 | 22-41 | 98-117 | 19-43 | 4-28 |
| **TM2** | 82-101 | 65-84 | 65-84 | 58-75 | 86-105 | 61-80 | 64-83 | 67-91 | 68-90 | 128-150 | 134-153 | 48-67 | 122-141 | 70-89 | 45-69 |
| **TM3** | 132-151 | 115-137 | 115-138 | 106-129 | 136-155 | 111-135 | 114-136 | 118-135 | 111-128 | 169-190 | 184-203 | 98-122 | 172-191 | 120-144 | 86-107 |
| **TM4** | 178-195 | 164-181 | 165-182 | 148-165 | 182-199 | 156-173 | 157-174 | 166-189 | 135-159 | 215-236 | 230-247 | 170-188 | 212-229 | 165-189 | 114-138 |
| **TM5** | 202-226 | 188-212 | 189-213 | 172-196 | 206-230 | 180-204 | 181-201 | 219-242 | 180-196 | 257-280 | 254-273 | 209-233 | 242-261 | 220-244 |  |
| **TM6** | 247-268 | 233-254 | 234-255 | 217-238 | 251-272 | 225-246 | 218-237 | 259-283 | 222-246 | 297-320 | 292-309 | 250-274 | 282-299 | 271-295 |  |
| **TM7** | 289-308 | 275-294 | 276-295 | 259-283 | 293-312 | 267-286 | 268-291 | 309-333 | 263-287 | 341-360 | 340-359 | 299-316 | 330-349 |  |  |
| **TM8** | 329-353 | 315-339 | 316-340 | 300-324 | 333-357 | 307-331 | 308-332 | 360-384 | 318-342 | 367-390 | 384-403 | 321-340 | 374-393 |  |  |
| **TM9** | 380-399 | 370-394 | 371-395 | 355-379 | 388-412 | 362-386 | 363-387 |  | 363-387 | 399-422 | 434-451 | 353-372 | 424-441 |  |  |
| **TM10** | 404-423 | 421-440 | 422-441 | 400-424 | 439-458 | 407-431 | 408-432 |  |  |  | 456-475 |  | 446-465 |  |  |
| **TM11** | 489-508 |  |  |  |  |  |  |  |  |  | 490-509 |  | 478-502 |  |  |
| **TM12** | 513-532 |  |  |  |  |  |  |  |  |  |  |  |  |  |  |
| **TM13** | 561-583 |  |  |  |  |  |  |  |  |  |  |  |  |  |  |
| **TM14** | 604-621 |  |  |  |  |  |  |  |  |  |  |  |  |  |  |
| **TM15** | 628-652 |  |  |  |  |  |  |  |  |  |  |  |  |  |  |
| **TM16** | 673-690 |  |  |  |  |  |  |  |  |  |  |  |  |  |  |
| **TM17** | 717-736 |  |  |  |  |  |  |  |  |  |  |  |  |  |  |
| **TM18** | 757-776 |  |  |  |  |  |  |  |  |  |  |  |  |  |  |
| **TM19** | 807-824 |  |  |  |  |  |  |  |  |  |  |  |  |  |  |
| **TM20** | 829-851 |  |  |  |  |  |  |  |  |  |  |  |  |  |  |
| **TM21** | 858-880 |  |  |  |  |  |  |  |  |  |  |  |  |  |  |
| **Length** | 904 | 454 | 455 | 438 | 472 | 446 | 446 | 398 | 401 | 444 | 527 | 388 | 517 | 304 | 160 |
